# The Cohort for Childhood Origin of Asthma and allergic diseases (COCOA) study: design, rationale and methods

**DOI:** 10.1186/1471-2466-14-109

**Published:** 2014-07-03

**Authors:** Hyeon-Jong Yang, So-Yeon Lee, Dong In Suh, Youn Ho Shin, Byoung-Ju Kim, Ju-Hee Seo, Hyoung Yoon Chang, Kyung Won Kim, Kangmo Ahn, Yee-Jin Shin, Kyung-Sook Lee, Cheol Min Lee, Se-Young Oh, Ho Kim, Jong-Han Leem, Hwan-Cheol Kim, Eun-Jin Kim, Joo-Shil Lee, Soo-Jong Hong

**Affiliations:** 1 Department of Pediatrics, Soonchunhyang University School of Medicine, Seoul, Korea; 2 Department of Pediatrics, Hallym Sacred Heart Hospital, Hallym University College of Medicine, Anyang, Korea; 3 Department of Pediatrics, Seoul National University College of Medicine, Seoul, Korea; 4 Department of Pediatrics, CHA Gangnam Medical Center, CHA University School of Medicine, Seoul, Korea; 5 Department of Pediatrics, Inje University Haeundae Paik Hospital, Busan, Korea; 6 Department of Pediatrics, Korean Cancer Center Hospital, Seoul, Korea; 7 Department of Pediatrics, Childhood Asthma Atopy Center, Research Center for Standardization of Allergic Diseases, University of Ulsan College of Medicine, 88 Olympic-ro 43-gil, Songpa-gu, Seoul 138-736, 760, Korea; 8 Department of Pediatrics, Yonsei University College of Medicine, Seoul, Korea; 9 Department of Pediatrics, Samsung Medical Center, Sungkyunkwan University School of Medicine, Seoul, Korea; 10 Department of Psychiatry, Yonsei University College of Medicine, Seoul, Korea; 11 Department of Rehabilitation, Hanshin University, Osan, Korea; 12 Institute of Environmental and Industrial Medicine, Hanyang University, Seoul, Korea; 13 Department of Food and Nutrition, College of Human Ecology, Kyung Hee University, Seoul, Korea; 14 Graduate School of Public Health, Seoul National University, Seoul, Korea; 15 Department of Occupational and Environmental Medicine, Inha University School of Medicine, Incheon, Korea; 16 Allergy TF, Department of Immunology and Pathology, Korea National Institute of Health, Osong Health Technology Administration Complex, 187 Osongsaengmyeong 2-ro, Yeonjae-ri, Osong-eup, Cheongwon, 363-951, Korea

**Keywords:** Cohort study, Gene-environment interaction, Allergy, Microbiota, Nutritional Status, Psychologic stress

## Abstract

**Background:**

This paper describes the background, aim, and design of a prospective birth-cohort study in Korea called the COhort for Childhood Origin of Asthma and allergic diseases (COCOA). COCOA objectives are to investigate the individual and interactive effects of genetics, perinatal environment, maternal lifestyle, and psychosocial stress of mother and child on pediatric susceptibility to allergic diseases.

**Methods/Design:**

The participants in COCOA represents a Korean inner-city population. Recruitment started on 19 November, 2007 and will continue until 31 December, 2015. Recruitment is performed at five medical centers and eight public-health centers for antenatal care located in Seoul. Participating mother-baby pairs are followed from before birth to adolescents. COCOA investigates whether the following five environmental variables contribute causally to the development and natural course of allergic diseases: (1) perinatal indoor factors (*i.e.* house-dust mite, bacterial endotoxin, tobacco smoking, and particulate matters 2.5 and 10), (2) perinatal outdoor pollutants, (3) maternal prenatal psychosocial stress and the child’s neurodevelopment, (4) perinatal nutrition, and (5) perinatal microbiome. Cord blood and blood samples from the child are used to assess whether the child’s genes and epigenetic changes influence allergic-disease susceptibility. Thus, COCOA aims to investigate the contributions of genetics, epigenetics, and various environmental factors in early life to allergic-disease susceptibility in later life. How these variables interact to shape allergic-disease susceptibility is also a key aim.

The COCOA data collection schedule includes 11 routine standardized follow-up assessments of all children at 6 months and every year until 10 years of age, regardless of allergic-disease development. The mothers will complete multiple questionnaires to assess the baseline characteristics, the child’s exposure to environmental factors, maternal pre- and post-natal psychological stress, and the child’s neurodevelopment, nutritional status, and development of allergic and respiratory illnesses. The child’s microbiome, genes, epigenetics, plasma cytokine levels, and neuropsychological status, the microbiome of the residence, and the levels of indoor and outdoor pollutants are measured by standard procedures.

**Discussion:**

The COCOA study will improve our understanding of how individual genetic or environmental risk factors influence susceptibility to allergic disease and how these variables interact to shape the phenotype of allergic diseases.

## Background

Allergic diseases such as asthma, atopic dermatitis, food allergy, and allergic rhinitis are some of the most common chronic diseases in the world [1]. In the past few decades, the global prevalence of these diseases rose abruptly and there is few signs that this trend is reversing itself. Indeed, in many parts of the world [1-3], particularly in Asia (including Korea) [4-7], the prevalence of allergic disease is continuing to rise. During the same period, Korea has experienced rapid economic growth marked by industrialization, modernization, urbanization, technological achievement, high education levels, and greatly improved living standards. This phenomenon is termed colloquially as the “Miracle on the Han River” [8] and has resulted in rapid changes in the residential environment and the population’s culture and dietary habits.

In human development, pregnancy is the most critical period in terms of function and structure. The developmental origins of health and disease (DOHaD) hypothesis [9] postulates that all organ systems undergo developmental programming *in utero* that shapes the physiology and metabolism of the adult. Indeed, there are multiple lines of evidence that suggest that exposure to various environmental variables *in utero* and during early childhood may play a major role in susceptibility to allergic diseases.

**Figure 1:**
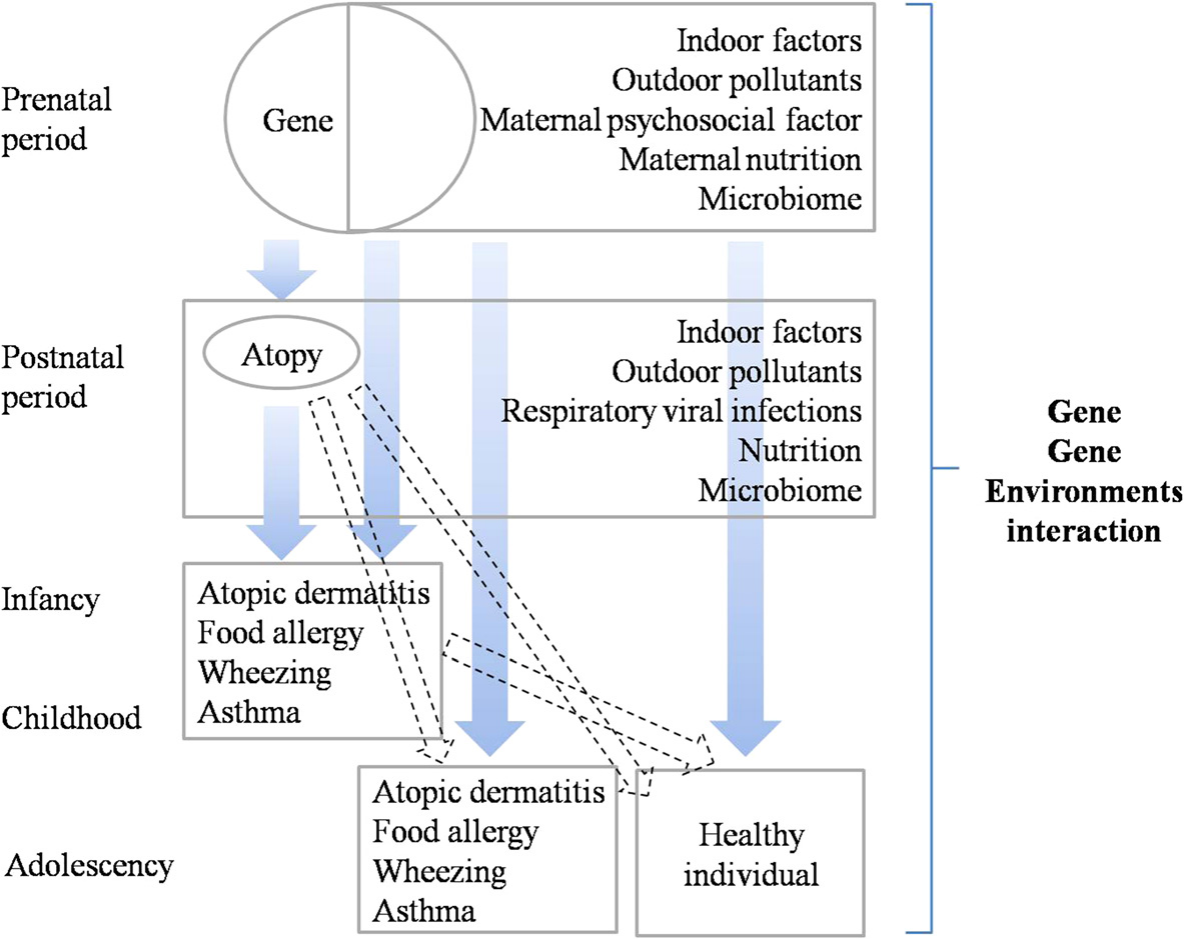
The COCOA study is designed to investigate the causal contribution of the following five environmental interventions to the development and natural course of allergic diseases: (1) perinatal exposure to indoor factors (namely, house-dust mites, bacterial endotoxins, tobacco smoking, and particulate matter 2.5 and 10), (2) perinatal exposure to outdoor pollutants, (3) perinatal maternal psychosocial stress, (4) perinatal nutrition, and (5) the perinatal microbiome.

Extensive epidemiological and laboratory studies have been performed to identify the environmental causes of allergic disease. Possible causes that have been studied include maternal factors before or during pregnancy such as disease, infection, diet, medication, and stress [10,11]. Various features of the indoor and outdoor environments in the prenatal and postnatal periods have also been examined. However, despite all this work, the etiology of allergic diseases remains unclear [12]. This may be due to the possibility that exposure to several environmental factors must occur before allergic disease is triggered. It may also be due to human genetic differences that shape the susceptibility of different populations or ethnicities to these triggers. Thus, susceptibility to allergic disease is likely to be influenced by the individual’s genetic background, maternal factors during pregnancy, and environmental factors in the perinatal period, and complex interactions between these factors. Since various factors, including race, culture, socioeconomic status, and place of residence, determine the degree of exposure to various environmental triggers, birth cohort studies are vital for identifying these gene-environment and environment-environment interactions [13]. Since Korea has a highly genetically homogeneous population that has undergone marked environmental changes recently, birth-cohort studies in this country are likely to yield interesting data that will illuminate the influence of rapid environmental deterioration on the development of allergic disease.The COhort for Childhood Origin of Asthma and allergic diseases (COCOA) birth-cohort study is currently underway. It is designed to investigate the causal contribution of the following five environmental factors to the development and natural course of allergic diseases: (1) perinatal exposure to indoor factors (namely, house-dust mites, bacterial endotoxins, tobacco smoking, and particulate matter 2.5 and 10), (2) perinatal exposure to outdoor pollutants, (3) perinatal maternal psychosocial stress, (4) perinatal nutrition, and (5) the perinatal microbiome. Thus, the COCOA study will help to delineate how these environmental factors interact with each other and the genetic background of the child during this critical time point in the child’s life (Figure 1). The study will consist of two investigation stages. The first will assess whether the perinatal factors act as individual risk factors for (a) the development of atopy, atopic dermatitis, food allergy, and recurrent wheeze in infancy, (b) the persistence of these diseases at the age of 10 years, and (c) the development of asthma and allergic rhinoconjunctivitis by the age of 10 years. The second stage will assess whether the ability of each perinatal variable to act as a risk factor for allergic disease is shaped by the other perinatal factors and/or the child’s genetics.

### Genetics

Genetic predisposition seems to be the biggest risk factor for allergic diseases. However, genes alone cannot explain the rising global prevalence of these diseases over the world because human evolution occurs over many centuries rather than over decades. Instead, it is likely that interaction between genes and the environment shape the phenotype of allergic disease. Supporting the role of the environment is that allergic diseases are more prevalent in developed countries than in developing countries. Moreover, the urban areas in Asian countries have higher prevalence rates of allergic diseases than rural areas [4,14]. Supporting the role of the genes is that Asian metropolises such as Seoul, Singapore, and Hong Kong in Asia still have relatively lower allergic disease prevalence and disease severity than metropolises in the United Kingdom and Australia [3]. The fact that a rapid change to Westernized culture and environments correlates positively with increased allergic disease prevalence but that differences remain between ethnic populations despite their similar environments suggests strongly that allergic disease susceptibility is shaped by interactions between genes and the environment. It is possible that when these interactions occur in the prenatal and/or postnatal periods, they induce epigenetic changes that later systematically influence the expression or suppression of genetic responses to environmental stimuli, thereby promoting or suppressing the development of allergic diseases [15].

In the COCOA study, the children will be bled at various ages and their exposures to various environmental factors at those ages will be measured. In this way, the epigenetic changes of the children at these ages can be assessed for correlations with specific environmental factors. Since epigenetic changes can pass into the next generation, such epigenetics research may help to identify the most effective long-term methods of preventing allergic diseases.

### The environment

The role of the environment in allergic disease has become the subject of increasing attention in the field of allergic disease management because its key role in the development of allergic disease suggests that it could be manipulated by preventive or therapeutic strategies. Air pollution resulting from industrialization, rapid urbanization, and the rising use of automobiles is of particular interest because the recent increases in allergic disease prevalence are largely attributed to these environmental changes. It is believed that air pollution may alter immune-programming *via* epigenetic mechanisms, especially in the prenatal and perinatal periods. Both outdoor and indoor air pollution are of interest. However, indoor pollution may have a greater influence on allergic-disease susceptibility because infants and young children spend most of their time indoors. In addition, indoor air pollutant concentrations can be 2 to 5 fold higher than outdoor levels. For this reason, the indoor and outdoor pollution exposure of the children will be measured at various ages in the COCOA study.

### Nutrition

Diet is arguably one of the most important environmental exposures in early life. The immune system is a extremely dynamic and metabolically active network that depends on dietary nutrients and its functions are highly sensitive to dietary changes [16]. Thus, dietary change is a logical candidate as a culprit responsible for the rise in allergic diseases. Supporting this is that the modern increase of caloric intake and dietary fat in the “Western diet” correlates with rises in prevalence of both metabolic and immune-mediated diseases [17]. By contrast, the Mediterranean diet associates with protection from childhood asthma and wheeze [18]. The protective elements within these diets are likely to include selenium, zinc, folate, vitamin E, and other antioxidants. However, specific supplementation with these molecules has had inconsistent and even paradoxical effects [19]. These inconsistencies may relate to complex interactions between specific nutrients and additional interactions with other modern environmental changes such as the human microbiome, stress, and air pollution. To identify these interactions, the COCOA study examines the maternal diet during pregnancy and the child’s diet at various ages.

### The microbiome

The “microflora hypothesis” proposes that, rather than specifically limiting infection, the hygienic western lifestyle limits general microbial exposure and thereby alters the colonization of the human gut, which in turn disrupts the appropriate development of immune systems and ultimately promotes the development of allergic diseases [14,15,20-23]. Infancy and early childhood have been identified as important periods for the development of gut microbiota and allergic diseases, respectively [24]. The emerging understanding of the importance of microbial contact during the vulnerable periods of fetal life, delivery, and infancy for healthy immune and metabolic programming creates new opportunities to improve infant health and reduce the risk of disease in later life. However, the underlying mechanism, the cause-effect relationship, and particularly the identification of helpful individual microbes and microbial compounds remain to be established and discovered. The hypothesis in the COCOA study is that microbial composition in early life is an important risk factor for allergic diseases in later life. To prove this, we will sample the fecal and skin microbiomes of the children at various ages and will seek to identify the factors that alter the microbial composition and function. The effect of changes in the microbiome on the metabolomics of the child will also be assessed.

### Maternal psychosocial stress and the child’s neurodevelopment

Psychological distress and allergic diseases show high comorbidity [25]. Moreover, there is evidence that psychological distress and allergic diseases have a bidirectional relationship [26,27]. A recent study showed that prenatal maternal distress promoted the development of allergic disease in the offspring [28]. However, the underlying mechanisms and a cause and effect relationship have not been elucidated, although there is some evidence that the underlying mechanisms may involve the hypothalamic-pituitary-adrenal axis [29].

In the COCOA study, to assess the interactive relationship between psychological distress and allergic diseases, the mother will be assessed for prepartum and postpartum depression and anxiety. The relationship between this stress and the development of allergic disease in the offspring will then be assessed. Furthermore, the child’s development and temperament and the presence of behavioral and psychological problems will be measured at various ages to determine whether it is influenced by maternal psychosocial stress and promotes the development of allergic disease. Whether the child’s neurodevelopment independently affects allergic disease susceptibility will also be tested. Thus, the COCOA birth-cohort study will comprehensively analyze the interactions between numerous factors within a genetically homogeneous ethnic population. COCOA will then be compared to other birth cohort studies in different ethnic populations with similar environments to further improve our understanding of the gene-environmental interactions in early life that promote or suppress the development of allergic diseases in later life. Ultimately, we expect that these investigations will provide better global strategies for the prevention and treatment of allergic diseases.

## Methods/Design

### Study population and setting

Seoul is the capital of South Korea and its largest metropolis: more than 10 million people (more than one-fifth of the total Korean population lives in Seoul) live in 605.41 square kilometers. Seoul has one of the highest population densities in the world (24,214/km^2^ in 2013 [30]): indeed, it is most densely populated metropolis of all the cities belonging to the Organization for Economic Co-operation and Development (OECD). Seoul also consists largely of a single ethnic population: 97.5% of its inhabitants have Korean ethnicity [31]. In addition, it is a highly developed city, ranking twelfth in the world in terms of gross domestic product (GDP). With a GDP of US$773.9 billion in 2012, it is also the fourth largest metropolitan economy after Tokyo, New York, and Los Angeles [32]. Finally, it is an extensively industrialized city that in 2013 had 2.8 million cars [33]. Thus, the Seoul population is an appropriate representative of Korean inner-city populations.

**Figure 2:**
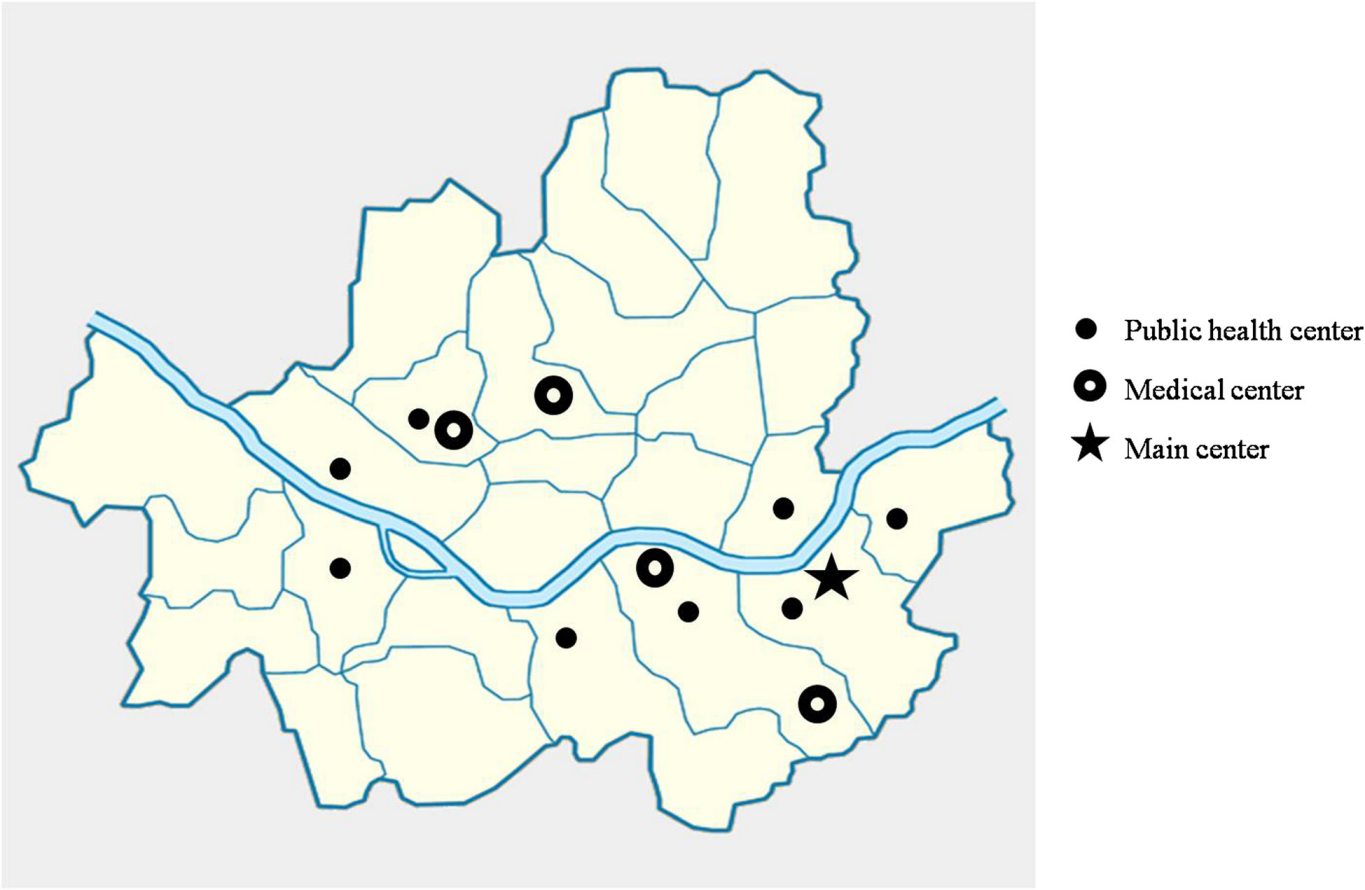
**The COCOA network consists of five medical centers (the Asan, CHA Gangnam, Samsung, Seoul National, and Severance Medical Centers) and eight Public Health Centers for antenatal care (Gandong-gu, Gangnam-gu, Gwangjin-gu, Mapo-gu, Seocho-gu, Seodaemun-gu, Songpa-gu, and Yeongdeungpo-gu).** All are located in Seoul, Korea.

The COCOA network consists of five medical centers (the Asan, CHA Gangnam, Samsung, Seoul National, and Severance Medical Centers) and eight Public Health Centers for antenatal care (Gandong-gu, Gangnam-gu, Gwangjin-gu, Mapo-gu, Seocho-gu, Seodaemun-gu, Songpa-gu, and Yeongdeungpo-gu). All are located in Seoul, Korea. Their geographical locations are provided by Figure 2. The main center is Asan Medical Center, which contains the genetics, epidemiology, environmental data constructing, *in vivo* and *in vitro* studies, nutrition, and psychology research teams.

### Study design

The COCOA study is a collaborative multi-center prospective birth-cohort study that is sampling nine regions of Seoul city to recruit a mother-child pair cohort that is representative of a Korean inner-city population. Its aims are to investigate whether genetics, maternal life-style, maternal and child’s psychological stress, and perinatal exposure to various environmental factors influence the development by the offspring of atopy and respiratory tract infections (RTI) early in life and allergic diseases in young adulthood. Whether and how these various factors interact with each other to shape susceptibility to allergic diseases will also be assessed. Recruitment of participant women and baby pairs started on 19 November, 2007 and will end on 31 December, 2015. The plan is to follow all trios of participant women, husband, and babe from before birth until the offspring is 10 years old. Thereafter, the study may be extended to follow up the offspring until they are 20 years of age.

Before commencement of the study, the study was approved by the Institutional Review Boards of Asan Medical Center (IRB No. 2008–0616), Samsung Medical Center (IRB No. 2009-02-021), Severance Medical Center (IRB No. 4-2008-0588), and CHA Gangnam Medical Center (IRB No. 2010–010).Seoul National University Hospital (IRB No. H-1401-086-550) joined the COCOA later and started recruiting in February, 2014. Written informed consent was obtained from each mother and their husband before the study-related interview was performed. The obtainment of consent was confirmed by each IRB.

### Study subjects (inclusion and exclusion criteria)

All eligible consecutive pregnant women who attend any of the five medical centers in Seoul (*i.e.* the Asan, Samsung, Severance, and CHA Gangnam Medical Centers, and Seoul National University Hospital) before 26 weeks of gestational age are asked whether they would like to participate in the COCOA study. The eligibility of the women is identified before recruitment. A woman is recruited if she lacks high-risk conditions that could affect the development of allergic disease in the child (*e.g.* diabetes, preeclampsia, anemia, and severe infections), plans to deliver at an affiliated medical center, and is a resident of Seoul city. The eligibility of the baby is determined by the collaborating obstetrician and pediatrician soon after delivery. Babies are excluded at birth if their gestational age is < 37 weeks or they have any major congenital anomalies or birth asphyxia requiring oxygen supplementation. The main purpose of the COCOA study was introduced with emphasis on their child’s general health to reduce the possibility to become high-risk cohort.

### Overview of the study process

This study is a longitudinal prospective birth-cohort study that will assess all offspring at birth and at 11 routine follow-up visits at 6 months and 1, 2, 3, 4, 5, 6, 7, 8, 9, and 10 years of age, regardless of whether the offspring develop allergic diseases. Clinical evaluations, including objective measurements of allergic diseases such as atopic dermatitis, food allergy, bronchiolitis, RTI, recurrent wheezing, asthma, and allergic rhino-conjunctivitis, are performed at each center. These clinical evaluations follow a standardized protocol (described in detail below). The delivery record of each infant is collected at birth in each center. Cord blood samples are collected from subjects at the time of delivery. Serial questionnaires are completed by the mother at enrollment and every scheduled visit to assess the baseline characteristics, nutritional status, psychological stress/development, development of allergic and respiratory illness, and exposure to various environmental factors. The study schedule is detailed in Table 1.

**Table 1:** Data collection schedule of the COhort for Childhood Origin of Asthma and allergic diseases (COCOA) study

					**Scheduled visits of the mother-child pair when the child is the indicated age**										
	**Enrollment (26**^**th **^**GA)**	**36**^**th **^**GA**	**Birth**	**1 mo**	**6 mo**	**1 yr**	**2 yrs**	**3 yrs**	**4 yrs**	**5 yrs**	**6 yrs**	**7 yrs**	**8 yrs**	**9 yrs**	**10 yrs**
Informed consent	**√**		**√**												
General questionnaire															
Maternal eligibility	**√**	**√**													
Familial demographics	**√**										**√**				
Familial socioeconomic status	**√**										**√**				
Familial history of allergic and respiratory diseases	**√**										**√**				
Maternal health questionnaires															
Maternal general health	**√**	**√**	**√**												
Maternal life style	**√**	**√**	**√**												
Maternal diet/nutrition	**√**	**√**	**√**												
Maternal psychosocial stress		**√**			**√**	**√**	**√**	**√**	**√**		**√**				
Child’s health questionnaire					**√**	**√**	**√**	**√**	**√**	**√**	**√**	**√**	**√**	**√**	**√**
Child’s general health					**√**	**√**	**√**	**√**	**√**	**√**	**√**	**√**	**√**	**√**	**√**
Child’s diet/nutrition					**√**	**√**		**√**			**√**		**√**	**√**	**√**
Child’s medication					**√**	**√**	**√**	**√**	**√**	**√**	**√**	**√**	**√**	**√**	**√**
Child’s allergic and respiratory illness					**√**	**√**	**√**	**√**	**√**	**√**	**√**	**√**	**√**	**√**	**√**
Child’s psychological development					**√**	**√**	**√**	**√**	**√**		**√**		**√**	**√**	**√**
Physician’s examination of child			**√**		**√**	**√**	**√**	**√**	**√**	**√**	**√**	**√**	**√**	**√**	**√**
Clinical measurement															
SCORAD index					**√**	**√**	**√**	**√**	**√**	**√**	**√**	**√**	**√**	**√**	**√**
Allergy skin test															
Mother					**√**										
Father			**√**												
Child								**√**			**√**			**√**	
Spirometryof child												**√**			
IOS of child									**√**						
FeNO of child										**√**					
Methacholine challenge test											**√**			**√**	
Respiratory virus (nasopharyngeal aspiration)			At every episode of respiratory viral illness												
Blood sampling															
Maternal blood					**√**										
Paternal blood			**√**												
Cord blood			**√**												
Child’s blood			**√**			**√**		**√**			**√**			√*	
Urine sampling of child								**√**			**√**			**√**	
Feces sampling of child				**√**	**√**			**√**			**√**				
Skin microbiome of child					**√**			**√**			**√**				
X-ray (bone-age) of child												**√**			
Questionnaire about the indoor environment															
Indoor environment assessment		**√**			**√**	**√**	**√**	**√**	**√**	**√**	**√**	**√**	**√**	**√**	**√**
Dust sample collection		**√**			**√**										
Indoor air-pollutant measurements		**√**													
Microbial sample collection		**√**			**√**										
Outdoor air-pollutant assessment		**√**			**√**										

*LH, FSH measurement at 9 years of age.

*Abbreviations*: *FeNO* Fractional exhaled nitric oxide, *FSH* Follicle-Stimulating hormone, *GA* Gestational age, *IOS* Impulse oscillometry, *LH* Luteinizing hormone, *SCORAD* Scoring atopic dermatitis.

### Data collection

#### Questionnaires

The mothers complete a modified version of the International Study of Asthma and Allergies in Childhood (ISAAC) questionnaire [34] at 36 weeks gestation. A series of questionnaires on confounding variables, maternal and child’s lifestyle (*i.e.* nutrition and psychological stress), and perinatal exposure to various indoor and outdoor environmental factors are obtained at 36 weeks gestation and every scheduled visit thereafter.

All mothers are asked to complete a questionnaire about their residential environment before and after the birth of their child. The following environmental factors are assessed: 1) residence characteristics (*e.g.* the type of building and floors, whether and when indoor renovation has been performed, whether and when the family have moved into a newly constructed house , *etc*.), 2) what type of stove is used for cooking and how the house is heated, 3) whether there are indoor domestic pets, 4) which organic chemical compounds (insecticide and detergent use) are used and when, and 5) whether the indoor air quality is affected by the presence of traffic roads near the home. Other factors that affect indoor air quality (*e.g.* parental smoking patterns) are also assessed. After birth, an additional questionnaire is given on a regular basis to determine the details of daycare center attendance, antibiotic use, and the development of respiratory symptoms of the offspring. This questionnaire has been validated previously and also includes questions about whether the child has eaten 113 food items during the preceding year. The amount of each food item that was eaten is measured by using nine non-overlapping intake frequencies (ranging from “rarely eaten” to “eaten more than three times per day”) together with three portion sizes (small, average, and large) [35]. The food and dietary intake is assessed by using a computerized nutrient-intake assessment software program (CAN-Pro 3.0; Korean Nutrition Society, Seoul, Korea) [36].

Maternal psychological distress is measured at 26, and 36 weeks of pregnancy, and at the time of birth. At the third trimester of pregnancy, the Center for Epidemiological Studies-Depression (CESD), State-trait Anxiety Inventory (STAI), Perceived Stress Scale (PSS), and Maternal Fetal Attachment Scale (MFAS) are used to measure maternal depression, anxiety, perceived stress, and maternal fetal attachment, respectively. After delivery, the Satisfaction with Life Scale, Korean Marital Satisfaction Inventory and Korean Parent Stress Index, as well as CESD and STAI, are used yearly to assess the maternal psychological state. The neurodevelopment of the child is evaluated by a series of questionnaires. The Korean Age and Stages Questionnaire, the Infant-Toddler Social and Emotional Assessment, and the Modified Checklist for Autism in Toddlers are completed at the scheduled visits. The infant’s temperament is assessed by using the Infant Behavior Questionnaire-Revised-Short Form, while the relationship between the parents and the child is measured by Parenting Relationship Questionnaire.

#### Blood samples for measuring gene expression and cytokine levels and to obtain DNA

The collaborating obstetricians are directly involved in the delivery and collect the cord blood at birth according to a standard protocol. Paternal blood is obtained at birth while maternal blood is obtained when the child is 6 months of age. Blood is also obtained from the child at the first, third, sixth, and ninth birthdays.

The cord blood is collected into a blood bag containing CPDA1 anticoagulant, after which the COCOA coordinator transports the bag to the laboratory in the main center. It is maintained at room temperature pending processing for less than 24 hours after collection until processing. The mononuclear cells in the cord blood are isolated by using the density gradient separation method and then cultured according to the method described by our previous study [37]. The culture supernatant is tested for the cytokine responses.

Plasma is generated from parental and child blood samples and analyzed at a central laboratory for the presence of antibodies to allergens. DNA from the cord blood sample and the peripheral blood samples of the parents and child are stored for subsequent genetic studies.

#### Urine analysis

Urine samples are collected at 36 weeks of pregnancy or before delivery from the mother and at the ages of 3, 6 and 9 years from the child. Midstream specimens are collected and subjected to routine urinalyses. At the ages of 3, 6, and 9 years, the concentrations of phthalate metabolites and cotinine in the urine are also measured. Phthalate metabolites are measured as described by the Center for Disease Control and Prevention Laboratory Procedure Manual [38].

#### Physician’s examination of the child

Health examinations of the child are performed at birth and each scheduled visit by pediatric allergy and pulmonology specialists at each center, who examine the child according to a standardized procedure for any abnormal signs such as throat infection, postnasal drip, erythema or bulging of the tympanic membrane, and wheezing. At the same visit, the body weight and height of the child are measured to the nearest 0.1 kg and 0.1 cm while the child wears light indoor clothing and without shoes. A certified nurses uses an automatic BP recorder to measure the child’s blood pressure (BP) a maximum of three times on the right arm while the child is seated and after the child has rested for 5 min.

#### Examination of the neuropsychological development of the child

At each follow-up visit, the psychological development of the child is evaluated. The free play, Mullen Scales of Early Learning, and Still Face procedures are conducted at 6 months of age. The strange situation procedures as well as free play and Mullen scales are conducted at 12 months of age. The Preferential Looking Paradigm, the Korean Wechsler Preschool and Primary Scale of Intelligence, and Korean Wechsler Intelligence Scale for Children-IV are employed at 2, 4, and 6 years of age.

#### Assessment of exposure to indoor and outdoor environmental factors

##### Outdoor air pollution

The concentrations of ambient air pollutants in various areas of Seoul at various times are compiled by using air monitoring data that are recorded routinely by monitoring stations operated by the Department of Environment, Republic of Korea. Each monitoring station measures the gaseous pollutants [nitrogen dioxide (NO_2_), ozone (O_3_), carbon monoxide (CO), and sulfur dioxide (SO_2_) and particulate matter (PM) hourly. These data are used to determine the daily, monthly, and yearly averages of these pollutants in the area around the monitoring station. These data in turn are used to estimate the exposure of the people living in the monitored area at the time of interest: for this, the kriging method using GIS (Geometric Information System) (Arc-Map, version 9.3; ESRI Inc., Redlands, WA, USA) is used [39]. However, land-use regression models will eventually replace the kriging method for estimating the level of exposure. Previous exposure values will be replaced by using this method.

##### Indoor air pollution

At 36 weeks of pregnancy, the indoor air pollutant concentrations in the mother’s residence are measured. The following will be measured: PM_10_ and PM_2.5_, biological aerosols (airborne bacteria and fungi), indoor aeroallergens (house-dust mite), and endotoxin. Mini-volume Air Sampler, Model 4.1 (Air metrics Co, USA) is used to sample particulate matters. Each sample collection is conducted in the mother’s room for 24 h at a constant sampling flow rate of 10 L/min. Samplers are positioned at least 1 meter away from the walls, ceiling, and door, and 1.2-1.5 meters above the floor. The particulate matters are measured by using a particle discriminator (Model GT-331, SIBATA Co., Japan) with a laser light-scattering optical particle counter. Microbial sampling is performed by using a Single Stage Air Sampler at the same place the particulate matters are sampled. Bacterial aerosols and airborne fungi are cultivated in Tryptic Soy Agar and Sabouraud Dextrose Agar for 24 hr at 24°C and room temperature, respectively. The colony-forming units (CFU) per m^3^ are calculated by dividing the CFU value by the indoor air volume. Dust samples are collected from a 1×1 m^2^ bedding surface for 2 min by using a vacuum cleaner in which the dust filter is fixed to allow even dust collection. After being put into Ziplock Bags to prevent the dust from being brushed off, the collected dust is kept cold (below −4°C) until analysis. Thereafter, 100 g and 50 g of each dust sample are used to measure house-dust mite and endotoxin, respectively as described elsewhere [40]. Briefly, *Der f* 1 (*Dermatophagoides farinae*) and *Der p* 1 (*Dermatophagoides pteronyssinus*) are measured by calorimetric analysis using an ELISA (Enzyme-Linked Immunosorbent Assay) Reader (Molecular Device, VersaMax). Endotoxin is determined by kinetic chromogenic analysis where a spectrophotometer is employed at a wave length of 405 nm according to the standard method described by the American Society for Testing and Materials [40].

#### Microbiome of the child and the indoor environment

Fecal and skin samples are obtained from the children at the age of 1 month, 6 months, 3 years, and 6 years of age. The microbiome sampling and DNA extraction methods have been described in detail elsewhere [23,41]. The microbiome of the indoor environment is also obtained at 36 weeks of pregnancy by the wiper method and from dust samples taken at the same time [42].

#### Outcome assessments

##### Skin prick tests

Skin prick tests (SPTs) are performed in the child on the third, sixth and ninth birthdays, in the father at the time of the child’s birth, and in the mother when the child is 6 months of age. ALK allergen solutions serve as allergen extracts and 10 mg/mL of histamine dihydrochloride and saline serve as positive and negative controls, respectively (Allergopharma, Reinbek, Germany) [43]. The longest length and width (measured perpendicular to the length at its midpoint) of the wheal are measured to the nearest millimeter after 15 min and are expressed as mean wheal size [44]. A child is deemed to have atopy when at least one common allergen evokes a positive response (wheal diameter ≥ 3 mm) and the mean wheal diameter to histamine exceeds 3 mm [45]. A child is deemed to be non-atopic when all common allergens yield a negative SPT response. The SPTs are performed with 18 common allergens for children (*i.e. Der f* 1, *Der p* 1, German cockroach, grasses mixture, alder, birch, oak, Japanese hop, mugwort, ragweed, dog epithelium, cat epithelium, *Alternaria alternata*, *Aspergilus fumigatus*, peanut, cow’s milk, egg white, and soybean), and 14 common allergens for parents (*i.e. Der f* 1, *Der p* 1, German cockroach, grasses mixture, Trees I, Trees II, mugwort, ragweed, dog epithelium, cat epithelium, *Alternaria alternata*, *Aspergilus fumigatus*, oak, and alder) [43].

##### Total serum IgE

Total serum IgE concentration in the child and their parents is measured by using a fluorescent enzyme immunoassay (AutoCAP System; Pharmacia Diagnostics AB, Uppsala, Sweden).

##### Respiratory infection and bronchiolitis

RTIs are defined as acute nasopharyngitis, rhinosinusitis, otitis media, croup, tracheobronchitis, bronchiolitis, or pneumonia. A child is deemed to have had an RTI when the mother responds positively to a series of questions about typical symptoms during each follow-up period or the family pediatrician who examined the child during the follow-up period has diagnosed RTI. Acute nasopharyngitis is diagnosed when there is an episode of runny/blocked nose or cough in the absence of other respiratory symptoms (*i.e.* no tachypnea, difficulty breathing, wheezing or rattly chest). Rhinosinusitis is diagnosed when two or more of the following symptoms that last at least 10 days occur during the follow-up period: anterior and/or posterior mucopurulent drainage, nasal congestion or blockage, cough, facial pain, pressure, or dullness, or a reduced or absent sense of smell [46]. Otitis media is diagnosed when there are signs and symptoms of inflammation in the middle ear [47]. Croup is diagnosed when there is an episode of the characteristic “barking” cough, hoarseness, and inspiratory stridor. Tracheobronchitis is diagnosed when its signs and symptoms, including a frequent, dry, hacking cough that lasts about 2–3 weeks, are preceded by upper respiratory infection symptoms such as rhinitis. Bronchiolitis is defined as wheezing [48] that is diagnosed by a physician during the follow-up period or by pediatricians and pediatric allergy and pulmonology specialists at the scheduled visits. Wheeze status is also assessed by asking the following at each visit: “Has he/she had wheezing or whistling in the chest at any time?” Pneumonia is diagnosed when its signs and symptoms are observed, including crackle, tachypnea, increased work of breathing accompanied by intercostal, subcostal, and suprasternal retractions, nasal flaring, the use of accessory muscles, cyanosis, and respiratory fatigue [49].

##### Food allergy

Food allergy is assessed at every scheduled visit by asking *via* a questionnaire whether a doctor had ever diagnosed food allergy in the offspring, whether food was suspicious, and whether medications had been prescribed for the condition. If there was a positive response, the diagnosis of food allergy was then confirmed by a pediatric allergy specialist during follow-up at each hospital. Moreover, the sera of the children taken at 12 months of age were tested for IgE antibodies specific for cow’s milk and egg white by using the Phadia ImmmunoCAP system (Phadia Diagnostics, Uppsala, Sweden). If the doctor believed that another food allergen was causing food allergy, antibodies against that food allergen were measured. The eligibility of the child for an open food challenge test was then determined on the basis of this information.

##### Atopic dermatitis

Atopic dermatitis is assessed at every scheduled visit by asking *via* questionnaire whether a doctor had ever diagnosed atopic dermatitis and whether mediations for the condition have been prescribed. When the infants are followed-up at the hospital, the presence of atopic dermatitis is clinically diagnosed by pediatric allergy specialists on the basis of the criteria of Hanifin and Rajka [50]. In children with atopic dermatitis, the physical examination conducted at the time of every-scheduled visits including standard scoring for signs of eczema using the Scoring Atopic Dermatitis (SCORAD) assessment [51].

##### Recurrent wheeze

Episodes of wheezing are identified by the questionnaire or the history taken by pediatric allergists. Recurrent wheezing is diagnosed when subjects have had three or more life-time episodes of wheezing, at least one of which has been confirmed by doctors.

##### Allergic rhinitis

Allergic rhinitis is suspected when the subject complains of relevant rhinitis symptoms (*i.e.* watery rhinorrhea, nasal obstruction, sneezing or itching). If a detailed history taken by doctors reveals that the subject has had allergic sensitization and presents with typical rhinitis symptoms for a sufficient period of time (for two or more consecutive days, and for more than 1 hour on most days), the subject is diagnosed with allergic rhinitis [52].

##### Bronchial hyperresponsiveness (BHR) and lung-function measurements

Pulmonary functions are assessed *via* various measures appropriate for the participant’s ability and age. Impulse oscillometry (at the age of 4) and exhaled nitric oxide analysis (at the age of 5) are conducted in a subset of participants who volunteer to undergo the analysis. All subjects undergo pulmonary function tests at the age of **7***via* conventional spirometry. BHR is assessed by measuring the concentration of methacholine that causes a 20% fall in FEV_1_ (PC_20_). When the PC_20_ is 16 mg/mL or less, the subject is diagnosed with BHR regardless of the clinical history assessed by the questionnaire.

##### Asthma

Asthma is assessed by various outcome measures. Typical asthma symptoms (*i.e.* wheezing, dyspnea, exercise-induced wheezing or nocturnal cough) are assessed by questionnaires on the regular visits at the ages of 6 months, 1 year, and then yearly thereafter when pediatric allergists also evaluate each subject on the basis of their detailed history and the physical exam. Asthma is diagnosed when subjects present a history of relevant asthma symptoms plus BHR or reversible airway obstruction. For preschool children who cannot conduct reliable lung function tests, subjects who have had three or more episodes of wheezing are deemed to have asthma if the pediatric allergists confirm this.

##### Management of study quality

The study data are managed by the Data Coordinating Center (DCC) that is located at Asan Medical Center. The questionnaires are gathered and encoded on optical mark recognition (OMR) cards by a trained clinical research center (CRC) nurse at each research site. The cards are then transferred to the DCC, where they are scanned and uploaded into the central database after CRC nurses at the DCC check for input errors. Follow-up calls are made by the data manager at DCC to verify data that are missing or contradictory. Personnel from the DCC visit each center yearly to review all aspects of data acquisition and transfer. The CRC nurses undergo bi-annual training sessions on the standard operating procedures (SOPs) for interviewing participants and coding data. As part of the quality control process, the database is subjected to periodic audits.

Biological samples are transferred and liquoted to the central laboratory immediately after the acquisition. After being appropriately treated, the samples are transferred to and stored at the Department of Immunopathology in the Korean National Institute of Health. The time spent transferring samples is minimized by using a specialized commercial service. The kinds and amounts of transferred samples are recorded on a separate management log at each site. For quality control and assurance, SOPs for collecting, processing and storing samples are established and followed.

#### Current status

**Figure 3:**
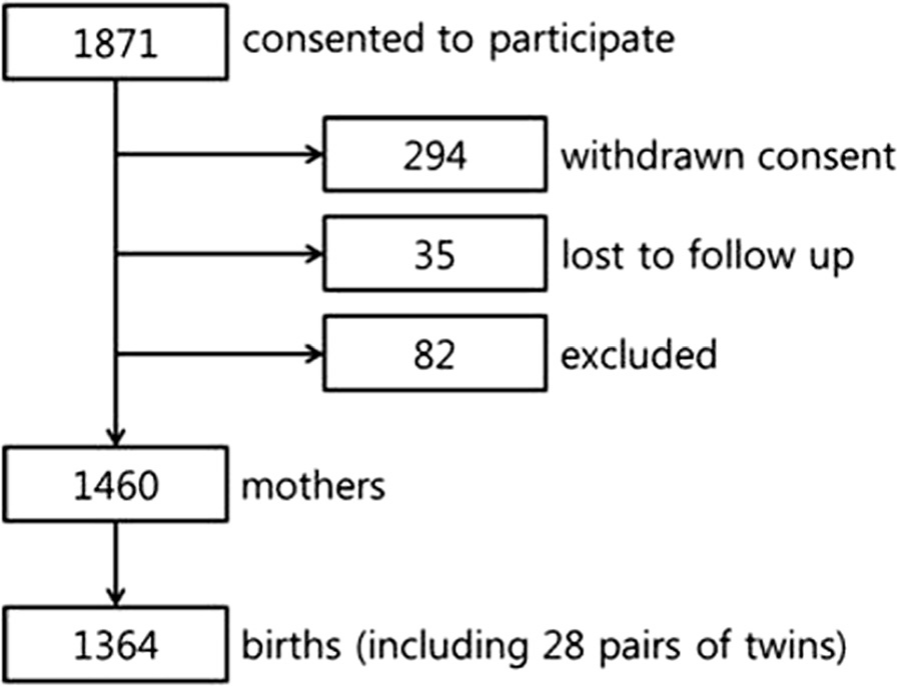
**Current status of the COCOA study.** A total of 1871 pregnant women have consented to participate in COCOA. Of these, 294 have withdrawn their consent and 35 have been lost to follow up. In addition, 82 women have been excluded, the most common reason being preterm birth. The remaining 1460 women have delivered 1364 infants, including 28 pairs of twins.

At the time of writing (May, 2014), 1871 pregnant women have consented to participate in COCOA. Of these, 294 have withdrawn their consent and 35 have been lost to follow up. In addition, 82 women have been excluded, the most common reason being preterm birth. The remaining 1460 women have delivered 1364 infants, including 28 pairs of twins (Figure 3).We expect to have 2500 pairs of pregnant women and offspring at the end of enrollment and that 250 children with asthma will be identified at 10 years of age.

## Competing interests

There are no financial or other issues that might lead to conflict of interests.

## Authors’ contributions

All authors contributed to and approved the final draft of the manuscript. Conception and design: KWK, KA, YHS, HK, HJY, JSL, and SJH. Analysis and interpretation: HJY, SYL, HYC, YJS and CML. Data collection and analysis: SYL, DIS, YHS, BJK, JHS, KSL, SYO, EJK, and JHL. Manuscript preparation: HJY, SYL, DIS, YHS, BJK, KA, YJS, HYC, CML, SYO, HK, JHL, HCK, and SJH. SJH and JSL contributed equally to this work and should be considered as co-corresponding authors.

## Pre-publication history

The pre-publication history for this paper can be accessed here:

http://www.biomedcentral.com/1471-2466/14/109/prepub
